# The gut virome in two indigenous populations from Malaysia

**DOI:** 10.1038/s41598-022-05656-3

**Published:** 2022-02-03

**Authors:** Chuen Zhang Lee, Muhammad Zarul Hanifah Md Zoqratt, Maude E. Phipps, Jeremy J. Barr, Sunil K. Lal, Qasim Ayub, Sadequr Rahman

**Affiliations:** 1 School of Science, Monash University Malaysia, 47500 Bandar Sunway, Selangor Darul Ehsan Malaysia; 2 Genomics Facility, Monash University Malaysia, 47500 Bandar Sunway, Selangor Darul Ehsan Malaysia; 3 Jeffrey Cheah School of Medicine and Health Sciences, Monash University Malaysia, Subang Jaya, Malaysia; 4 School of Biological Sciences, Monash University, Melbourne, VIC 3800 Australia; 5 Tropical Medicine and Biology Multidisciplinary Platform, Monash University Malaysia, Subang Jaya, Malaysia

**Keywords:** Phage biology, Virology, Microbiology, Microbial communities, Metagenomics, Microbiome

## Abstract

The human gut contains a complex microbiota dominated by bacteriophages but also containing other viruses and bacteria and fungi. There are a growing number of techniques for the extraction, sequencing, and analysis of the virome but currently no standardized protocols. This study established an effective workflow for virome analysis to investigate the virome of stool samples from two understudied ethnic groups from Malaysia: the Jakun and Jehai Orang Asli. By using the virome extraction and analysis workflow with the Oxford Nanopore Technology, long-read sequencing successfully captured close to full-length viral genomes. The virome composition of the two indigenous Malaysian communities were remarkably different from those found in other parts of the world. Additionally, plant viruses found in the viromes of these individuals were attributed to traditional food-seeking methods. This study establishes a human gut virome workflow and extends insights into the healthy human gut virome, laying the groundwork for comparative studies.

## Introduction

Viruses are the most abundant biological entity on earth, with estimates of 10^31^ viruses residing around the world^1^. Being omnipresent, viruses inhabit many different environments including water, soil, air, and biological entities, such as humans^2^. Viruses are generally much smaller than bacteria^3^ and are notorious for having caused pandemics throughout human history, such as those for smallpox, measles, Ebola, influenza H1N1, and the current COVID-19 pandemic. Although such pathogenic viruses get much attention, other types of viruses such as bacteriophages are important research tools, enabling a deeper understanding of basic cellular processes, microbial ecology, and the therapeutic potential of viruses.

Earlier studies estimate that the human gut viral density lies between 10^9^ and 10^12^ particles per gram of faeces^3,4^. Most viruses in the gut are represented by bacteriophages, with a small minority belonging to eukaryotic viruses (< 5%)^5^. Further, it is estimated that less than 1% of the virome has been sequenced and identified, suggesting that the majority of the virome has yet to be characterized^6^. Human gut virome studies have recently expanded substantially, however as far as we are aware, only one study has used long-read sequencing technology to characterize the human gut virome^7^. The study uses an amplification approach to achieve the required amount of DNA, which has been shown to induce bias in the abundance of different viral species^8^.

Previous studies of the adult gut virome have consistently revealed longitudinal stability of the composition and diversity of viral species^9^. For instance, a case study on a single individual for 2.5 years demonstrated that 80% of the 478 viral contigs were retained throughout the study period^10^. Additionally, another study discovered that the gut virome of 10 healthy adults were distinct and remained stable through a one-year period, in terms of relative abundance, richness, and viral loads of bacteriophages^9^. However, most human gut virome studies are focused on the United States and the only Asian country with human gut virome studies is China^5^.

Healthy gut viromes need to be characterized prior to examining disease-related virome changes. However, the range of variation of healthy viromes is unknown as only a few studies on the healthy gut virome have been carried out. None of these have focused on Southeast Asian populations, particularly indigenous populations, which may show unusual variation due to their lifestyles often being very different to mainstream populations.

In east Malaysia, it was estimated in 2015 that out of about 26 million people, the indigenous people, Orang Asli (OA) comprise 0.5% (150,000) of the population^11^. Within OA, the three largest divisions include Proto-Malay (sub-tribes: Orang Seletar and Jakun), Senoi (sub-tribes: Mahmeri and Semai), and Negrito (sub-tribes: Jehai, Mendriq and Batek). The OA are diverse both genetically^12^ and in their lifestyles. The Jakun and Jehai differ in their degree of assimilation to the mainstream lifestyle in Malaysia and the viromes of individuals from these tribal groups have not been previously characterized. The Jakun sampled are a group of semi-urban individuals from South Malaysia in Segamat, Johor, Malaysia from the Proto-Malay group. The Jehai sampled are a forest dwelling community inhabiting the northern parts of the peninsula in the state of Perak and Kelantan and are from the Negrito group.

Comprehensive studies of the gut virome from genetically, geographically, and economically diverse but healthy individuals are crucial in providing a better estimate of the virome composition among apparently healthy individuals with consideration to the effects of these variables on the healthy gut virome. The identification of healthy gut virome may help develop therapeutic approaches that involve maintaining a healthy gut microbiome. For example, one option for the treatment of *Clostridioides difficile* infection is faecal microbiota transplantation^13^ and knowledge of the recipient gut virome may be crucial in the maintenance of transplanted microbiome.

The study of the human gut virome comes with a set of challenges. Currently, virome DNA extraction methods have not been standardized, and DNA yield ranges are typically low, below 50 ng per gram of faecal sample^14^, depending on isolation and extraction steps. Furthermore, some methods, such as selective filtration and density gradient methods, are selective and only enrich for viruses of a particular density^14^. An alternative is to use DNA amplification methods, but this brings its own set of problems including biased amplification and the formation of chimeras^15,16^. Additionally, once sequences are obtained it may be difficult to identify them as viral, given the vast variability in viral sequences and the small proportion of viruses that have been described. One can show that sequences are non-human or non-bacterial but positive identification as viral can be problematic. This problem is especially acute when an under-researched group like the Malaysian Orang Asli are being researched. This is because almost all work on the virome has been on Western populations^5^ and the virome of populations differing in both the genetics and the diet may well contain a large proportion of viruses never before reported. To get around this problem of positive identification of viral sequences, a number of bioinformatics approaches can be taken but perhaps the most promising is by translation of the DNA sequences obtained into proteins and then searching for similarity with known viral proteins. The advantage of long DNA reads in this case is that if one of the encoded proteins is recognised then the whole contig can be regarded as being of viral origin.

To sum up, this paper addresses a number of difficulties that are faced in virome research. Identification of the gut virome is dependent on the sequencing technology used. This virome study is among the first to utilize long-read sequencing for the study of the human gut virome in southeast Asia. Long-read sequencing provides a better resolution of the virome, but requires a larger DNA input per sample than conventional short-read sequencing. Therefore, there is the requirement for the development of a suitable and effective method to yield sufficient DNA from limited faecal samples for the long-read sequencing approaches. In this study, we report the optimization of a virome DNA extraction protocol from faecal matter and an analysis pipeline of the sequences obtained. We apply this workflow to the analysis of samples from Jakun and Jehai individuals in Malaysia to expand our knowledge of the human virome.

## Results and discussion

### Viral DNA extraction

In previous studies, the range of yields of virome DNA extraction methods were low, typically below 50 ng per gram of faecal sample^14^. Thus, selecting a simple yet effective method is crucial for better characterizing the human gut virome. In this study the method selected for optimization was a method that utilized PEG precipitation, herein referred to as PEG-method, based on the report by Shkoporov and colleagues^17^. Briefly, homogenized faecal samples in buffer were filtered twice through 0.45 μm filters, followed by precipitation of viral particles with PEG. Viral particles were treated with DNase and RNase and lysed with proteinase K. Lysates were extracted with Phenol:Chloroform:Isoamyl Alcohol (PCIA) and purified using the DNeasy Blood and Tissue Kit following the manufacturer’s protocol. This method was selected due to its simplicity, cost efficiency, and low levels of bias with the aim of producing sufficient viral matter (estimated to be > 100 ng per gram) of faecal matter for long-read sequencing.

Initial extractions were carried out on a mock virome, consisting of *Escherichia virus T4*, *Escherichia virus Lambda* and *Escherichia virus T3* phage. After consistently achieving 600 ng of DNA per gram of the mock virome, the method was applied on human faecal samples. DNA yield increased to an average of about 350 ng of DNA per gram of faecal sample in 60 μl of elution buffer (approx. 20 ng/μl) (see Supplementary Fig. S1 online), through a series of optimizations. These included increasing DNase and RNase concentrations to 10 U per sample each and incubation times to 2 h. Additionally, proteinase K incubation time was increased to 2 h and a step pooling three sets of 3 g of faecal material per sample from an individual was introduced. Thus, we established an amplification-free efficient virome DNA extraction protocol by optimizing an existing method with PEG precipitation (see Supplementary Fig. S2 online). The effectiveness of PEG-method supports a recent study that came to the same conclusion, suggesting that PEG is very suitable for use for virome DNA extractions in terms of cost and efficiency, providing relatively high yields of DNA and good purity of DNA compared to other methods^18^.

### Viral prediction

Long-read sequencing was performed on faecal samples from six Orang Asli females (three Jakun and three Jehai). The number of reads from each sample varied greatly (Jakun: 20,118–289,003, Jehai: 47,072–375,911), which consequently affected number of contigs assembled by the program metaFlye^19^ (Jakun: 3–187, Jehai: 13–766). The average N50 (defined so that half the total nucleotides of an assembly belong to contigs of this length or longer) was 28,724 bp for the Jakun and 47,727 bp for the Jehai samples. The program VirSorter^20^ was used to predict viral contigs from six long-read sequences. In total, 61% (Jakun: 59%, Jehai: 62%) of microbial sequences were assigned as viral through VirSorter, suggesting that more than half the sequences were viruses. Additionally, when aligning these sequences to bacterial marker genes (using the program ViromeQC^21^) and human reference genome (version GRCh38), percentage of alignment was 0.54% (Jakun: 0.60%, Jehai: 0.48%) and 0.08% respectively. The remaining ~ 38% of sequences may be attributed to viruses of unknown bacterial or eukaryotic origin, suggesting these could lie within the viral dark matter. Viral dark matter comprises viral sequences that have not yet been identified, or are unknown contaminants. In previous virome studies such as human gut virome and ocean water virome, the amount of viral dark matter ranged from 40 to 90%^22^. Therefore, the amount of viral dark matter in this study is relatively low, but within expectations.

### Jakun and Jehai Orang Asli viromes

The virome from individuals belonging to a semi-urbanised Jakun Orang Asli and a hunter-gatherer Jehai Orang Asli tribe from Peninsular Malaysia were characterized in this study. The analysis utilized metaFlye^19^ assembler program, followed by VirSorter^20^ virus identification, then the sequences were analysed in parallel with both the vConTACT2^23^ taxonomic assignment program and the Genome Detective Virus Tool (GDVT)^24^.

**Figure 1:**
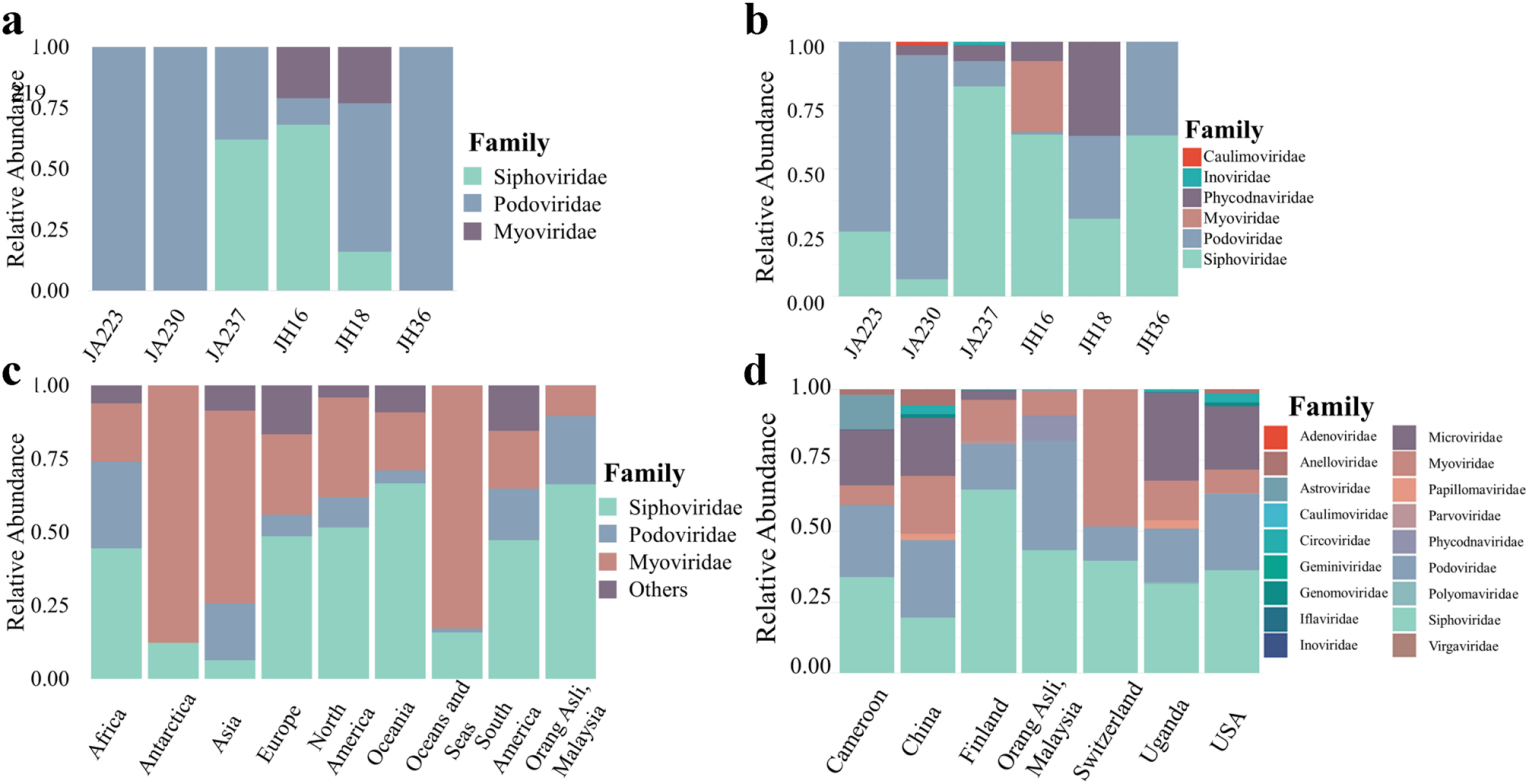
Relative abundance plot of viral families. (**a**,**b**) Six individuals, three from Jakun Orang Asli (JA223, JA230, JA237) and three from Jehai Orang Asli (JH16, JH18, JH36), taxonomy assigned by vConTACT2 and GDVT respectively. (**c**) Caudovirales order among Orang Asli, Malaysia, (n = 6) against different continents in isolates from NCBI Virus Database, Africa (n = 484), Antartica (n = 73), Asia (n = 8322), Europe (n = 6815), North America (n = 33,966), Oceania (n = 241), Oceans and Seas (n = 1052), South America (n = 161). (**d**) Orang Asli from Malaysia against Human Gut Virome Database separated based on country of origin, Cameroon (n = 29), China (n = 44), Finland (n = 56), Orang Asli (n = 6), Switzerland (n = 8), Uganda (n = 65), and USA (n = 279).

Viral taxonomic assignments were assigned to all samples using vConTACT2 and plotted as a relative abundance plot (Fig. 1a). It was apparent that three viral families, *Myoviridae*, *Podoviridae* and *Siphoviridae*, dominated the virome. All three viral families originate from the same viral order, *Caudovirales*. Surprisingly, only Jehai samples were found to contain the viral family *Myoviridae*. Besides that, the abundances were not highly distinguishable between Jakun and Jehai.

GDVT recovered a more diverse set of viral families (Fig. 1b). Consistently, the viromes of all individuals were dominated by the *Caudovirales* order, namely *Myoviridae*, *Podoviridae* and *Siphoviridae* viral families. However, all samples appear to have an increased assignment of *Siphoviridae* viral family with GDVT, perhaps due to the different database used as reference. Besides, the *Myoviridae* viral family which was found only in Jehai with vConTACT2, was also observed at low abundance in the Jakun with GDVT. The difference in the two tools may be attributed to the difference in database and techniques used to assign taxonomy, for example, some viruses present in one database that are not present in the other may have cause dissimilarities in the findings.

Interestingly, two samples had sequences attributed to plant viruses from the *Caulimoviridae* and *Phycodnaviridae* family. *Phycodnaviridae* has previously been reported in the infant gut virome^25^ and human oropharyngeal virome^26^, suggesting that it is highly likely that plant viruses can occasionally exist within the human gut depending on the diet of individuals. *Phycodnaviridae* have been reported to replicate within cells such as Chlorella^27^ A recent study stated that although Jehai Orang Asli do buy food, their purchasing power is low due to their socioeconomic status^28^.Therefore, they also rely on traditional food seeking practices to meet their dietary requirements. Interestingly, the same individual with the *Phycodnaviridae* viral family was predicted to have a large proportion of *Tubulinea*, which are a group of eukaryotic organisms including amoeba (see Fig. 4 later), suggesting potential amoeboid infection. The presence of *Phycodnaviridae* and *Tubulinea* in this individual may reflect the use of contaminated water sources and unsanitary living conditions and hence the origin of *Phycodnaviridae* viruses in these individuals may likely be from consumption of contaminated food or water.

Additionally, we observed the viral family of *Inoviridae* in one sample. This is a single stranded DNA (ssDNA) bacterial virus which would not have been detected through conventional short-read sequencing. For ssDNA viruses to be detected by short-read sequencing an amplification step is required, which may introduce amplification bias. The practicality of long-read sequencing to identify ssDNA viruses has also recently been demonstrated by Ji and colleagues^29^, as MS2 (ssDNA of the family *Leviviridae*) and PhiX174 (ssDNA of the family *Microviridae*) were both successfully classified with long-read sequencing.

Taxonomic assignments with both vConTACT2 and GDVT show that the most abundant viral order in the Orang asli gut virome is *Caudovirales*, which includes families *Podoviridae*, *Siphoviridae*, *Myoviridae* among others. This result supports previous virome findings that most sequences consist of the order *Caudovirales*^9^. In addition, the presence and abundance of viral sequences were different across all six samples, suggesting a high interpersonal variability in the human gut viromes of these indigenous populations (Fig. 1b).

The noteworthy difference between the Orang Asli groups was the richness of viral family *Podoviridae* and the scarcity of viral family *Myoviridae* in the Jakun compared to the Jehai (see Supplementary Fig. S3 online). However, it should be noted that the small sample size hinders any statistical significance from being assigned to these differences in the gut viromes of the two communities. Despite that, these findings are suggestive evidence that there might be virome differences between the two populations.

During finalization of this manuscript, we became aware of Luis et al.^30^ who described a new viral database, the Gut Phage Database and their finding that most of the viruses belong to p-crAssphage which has been reported to possess a morphology similar to *Podoviridae* family^31^. The applicability of their findings to the Orang Asli we have described require further investigation.

### Malaysian Orang Asli and worldwide viromes

Considering the high levels of viral order *Caudovirales* among the Orang Asli population in this study, the taxonomy of the Orang Asli virome was compared with viral families from the viral order *Caudovirales* deposited in the NCBI Virus Nucleotide Database (Fig. 1c) as of 17th January 2020 and stratified by the continent of origin. Viral families *Siphoviridae*, *Podoviridae*, and *Myoviridae* dominated regardless of the continent of origin. However, the abundance of each viral family varied drastically depending on continent, suggesting a possible role of geographical effects on dissimilar abundance of viral families.

Additionally, the taxonomy of the Orang Asli viromes (this study) assigned by GDVT were also compared to the Human Gut Virome Database (HGVD) (Fig. 1d), which comprises metagenomic human gut virome studies compiled from several countries. Surprisingly, the Orang Asli groups did not contain any of the ssDNA viral family *Microviridae*, which is in considerable abundance in several countries, most notably Cameroon (20%), China (20%), Uganda (30%), and USA (22%) (Fig. 1d). This may indicate that *Microviridae* could be less prevalent in indigenous Malaysian populations compared to other populations. However, previous studies utilized multiple displacement amplification (MDA) that preferentially amplifies ssDNA, suggesting a possible bias in frequency of ssDNA from metagenomics studies^32^. The family *Phycodnaviridae* which was found in this study, was absent in the HGVD, suggesting that it may be region specific and hence, perhaps explains not identifying it when using vConTACT2 to characterize the viral sequences.

**Figure 2:**
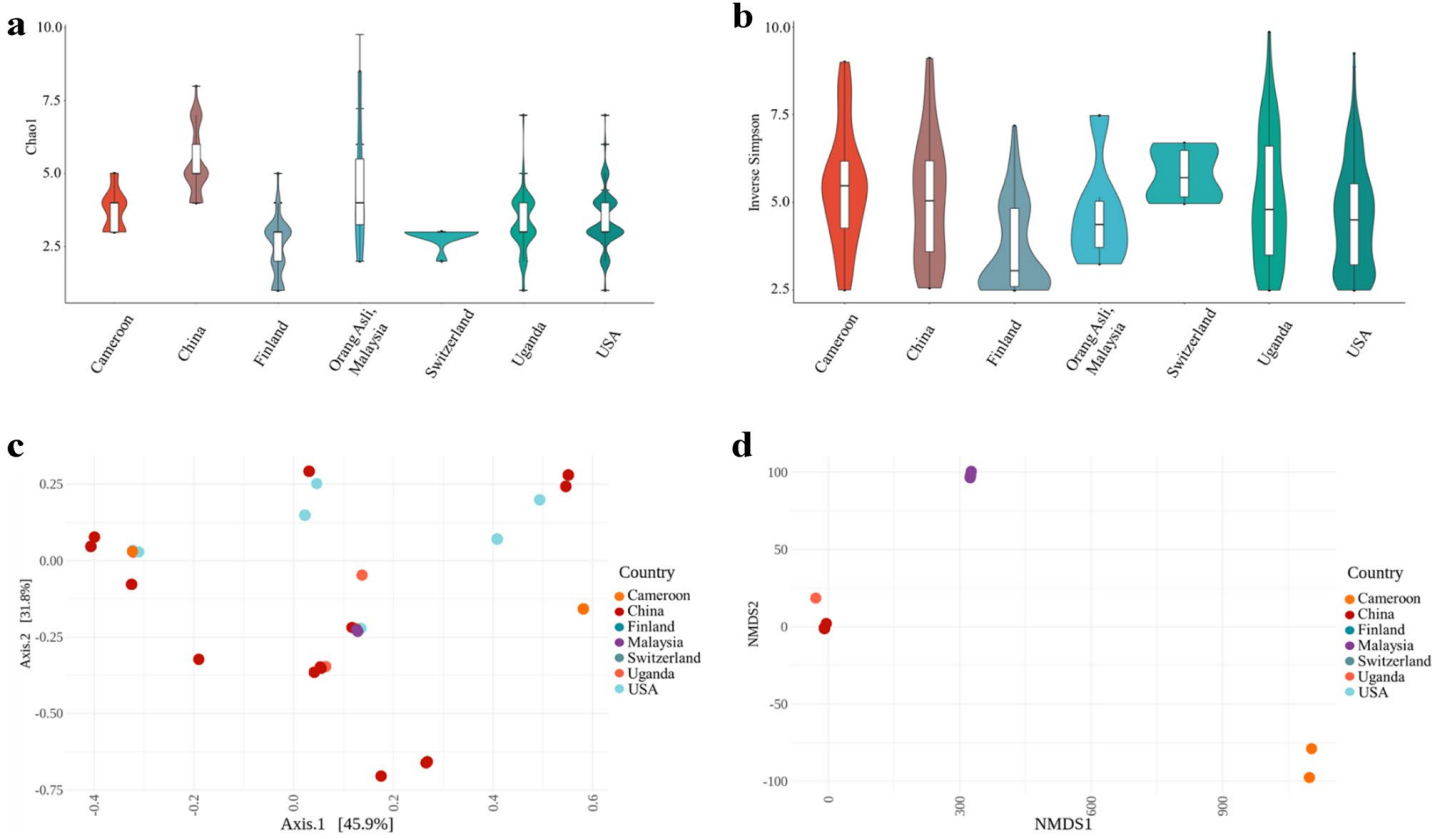
Diversity metrics for Orang Asli from Malaysia and worldwide metagenomics virome data from HGVD. (**a**,**b**) Viral alpha diversity. The boxplots show the alpha diversity of the viral communities in various countries compared to Orang Asli from Malaysia by means of viral sequences using alpha-diversity indexes; (**a**) Chao1 and (**b**) Inverse Simpson. (**c**,**d**) Viral beta diversity. The two-dimensional scatter plot shows the beta diversity of viral compositions in various countries, Cameroon (n = 29), China (n = 44), Finland (n = 56), Switzerland (n = 8), Uganda (n = 65), and USA (n = 279) compared to Orang Asli, Malaysia, (n = 6) generated by ordination methods; (**c**) principal coordinate analysis (PCoA) and (**d**) Non-metric multidimensional scaling (NMDS) from Bray–Curtis distance matrix.

Alpha diversity measures the number of species within a given area or ecosystem. The alpha diversity measures used here are estimators (Chao1 and Inverse Simpson index) of diversity based on the abundance and number of species. Despite the variations in viral families, there was no significant difference in alpha-diversity between any countries compared here (Fig. 2a,b; see Supplementary Table S1 online), suggesting that virome richness remains consistent irrespective of geographical region. Beta diversity describes how much of the total diversity observed within a group or set is due to a particular member of a set. Thus Fig. 2c,d display the beta-diversity of the named country relative to the total diversity of the set composed of the named country and the Orang Asli. The further the named country samples are from 0,0 the more different the viruses are in that country are from the ones found in the Orang Asli. Beta-diversities between all the countries and the Orang Asli samples were tested using permutational multivariate analyses of variance (PERMANOVA) using Bray–Curtis distance matrices (Fig. 2c,d) and having a significance value of *p* < 0.001 in all cases (see Supplementary Figs. S4 and S5 online). It was found that the virome beta-diversity of Orang Asli, Malaysia group from this study was significantly different to all other countries in HGVD, indicating that the Orang Asli, Malaysia virome compositions were highly dissimilar compared to virome compositions identified from other countries. However, after carrying out analysis of the dispersion of the samples, a possible confounder of the geographical effects found here, it was seen that the Switzerland samples had a significantly different dispersion to all other samples and so differences observed in PERMANOVA with the Switzerland samples cannot be relied on (also USA-Cameroon; Supplementary Fig. S11 online).

### Putative viral hosts

Putative hosts for viral sequences were determined by GDVT (Fig. 4 and Supplementary Fig. S6 online) with 67% of viruses assigned a potential bacterial or eukaryotic host. The abundance of viral host was approximated by the abundance of each virus. In total 33% of the viruses had an unknown host. The most abundant putative host among all samples were from the *Firmicutes* phylum, with 60% of Jakun Orang Asli and 28% of Jehai Orang Asli viromes being assigned to this phylum. Additionally, *Bacteriodetes* and *Proteobacteria* hosts are also deduced to contribute 4% and 6%, respectively, of the gut viromes in the Orang Asli.

### Functional profile of Orang Asli viromes

To functionally profile the gut DNA virome, viral sequences were annotated using the program DRAM^33^. Open reading frames were screened to determine viral genes and assigned classifications, summarized in Table 1 as either viral hypothetical genes, viral genes with unknown function, viral genes with host benefits, viral genes with viral benefits, viral replication genes, or viral structure genes based on VOGDB functional classification (https://vogdb.org).

**Table 1:** Functional categories of genes among the two Orang Asli populations.

Population	Viral genes							Total gene count
	Hypothetical	Unknown functions	Host benefits	Viral benefits	Replication	Structural	Total	
Jakun	1598	105	27	0	13	9	1752	1752
Jehai	11,849	465	204	5	37	16	12,562	12,576

Predictably, the lack of knowledge on the virome contributed to the large proportion of hypothetical genes, amounting to 91% and 94% among Jakun individuals (n = 1598) and Jehai individuals (n = 11,849) respectively. Furthermore, the category of viral genes with unknown functions yielded 105 genes (5.99%) in Jakun individuals and 465 genes (3.70%) in Jehai individuals. This proportion of hypothetical and unknown genes demonstrates the need for a thorough investigation on the human gut virome, including the use of other omic approaches such as metatranscriptomics, metaproteomics and metabolomics to gain a better grasp of unknown and hypothetical genetic factors of microbes as well as the human host. Conversely, 27 genes (1.54%) in Jakun and 204 genes (1.62%) in Jehai viromes were designated as viral genes with host benefits, which are important for the evolution and persistence of the host. For instance, the gene anaerobic ribonucleoside-triphosphate reductase (nrdD) was found in a *Myoviridae* virus, which is responsible for the catalysis of ribonucleotides into deoxyribonucleotides, essential for DNA synthesis and repair in bacteria. Similarly, Integrase genes (int) are important for gut microbiome homeostasis because they allow viruses to transfer genes to bacteria which may enhance bacterial metabolic functions, eliminate competing organisms, or increase virulence^34^. For example, some viruses enhance bacterial metabolic functions by having the ability to promote biofilm formation in bacteria, subsequently improving bacteria fitness^35^.

**Figure 3:**
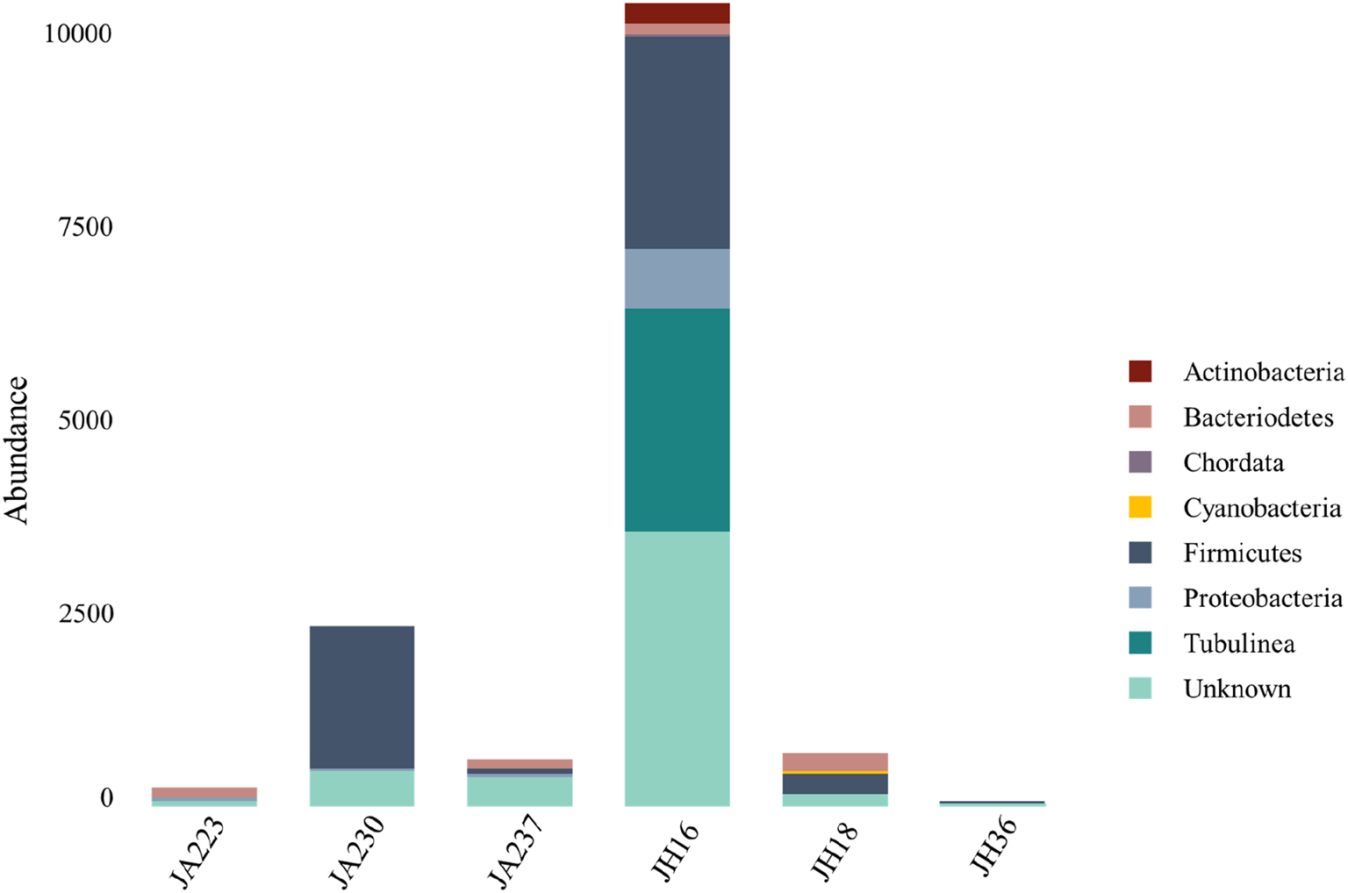
Abundance of putative hosts in six samples. Three individuals from Jakun Orang Asli (JA223, JA230, JA237) and three individuals from Jehai Orang Asli (JH16, JH18, JH36) taxonomy assigned from GDVT, expressed as counts of putative bacteria depending on viral abundance.

**Figure 4:**
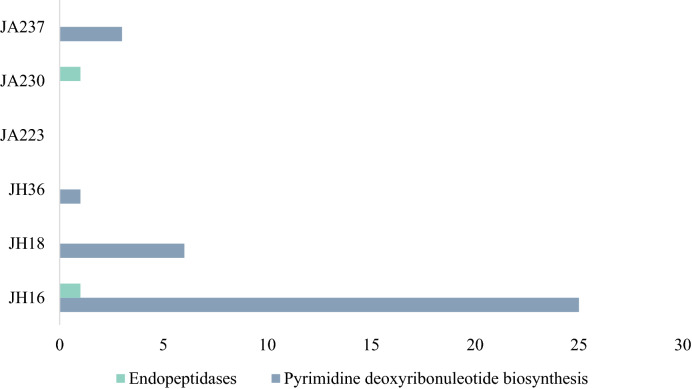
Auxiliary Metabolic Genes in the Virome of six Orang Asli. Three individuals from Jakun Orang Asli (JA223, JA230, JA237) and three individuals from Jehai Orang Asli (JH16, JH18, JH36).

Viral genomes often also acquire host metabolic genes, known as auxiliary metabolic genes (AMG), which could improve viral fitness and promote viral reproduction. AMGs were identified for all samples and are summarized in Supplementary Table S4 online. AMG modules found in more than two samples were Endopeptidases and Pyrimidine biosynthesis (Fig. 3). Endopeptidases act as an endolysin at the end of an infection cycle^36^, hence the detection suggests a presence of lytic bacteriophages in the human gut. The presence of pyrimidine biosynthesis has previously been reported in marine viruses and is found to be highly important in viral replication^37^. Additionally, host cells (bacteria and eukaryotic cells) may need to utilize pyrimidine biosynthesis pathways from viruses to supply sufficient nucleoside 5′-triphosphate (NTP) for fast-dividing cells such as the intestinal epithelial cell lining the human gut and fast-replicating viruses^38^. AMGs involved in the pyrimidine pathway that are found in this study are dUTP pyrophosphatase (dUTPase) and ribonucleoside reductase (RNR). dUTPase prevents misincorporation of uracil^39^ and is crucial in maintaining accurate viral genome replication and has been found in many viral genomes such as Epstein-Barr virus^40^, Feline immunodeficiency virus^41^, and African swine fever virus^42^. RNR is responsible for changing the ribonucleotides into deoxyribonucleotides, which are necessary for DNA synthesis^43^. Interestingly however, Enav and colleagues^37^ found that both purine and pyrimidine biosynthesis pathways were enriched in the AMGs, whereas in this study only pyrimidine biosynthesis were annotated (Fig. 4).

### Sequencing platform validation

We compared two sequencing platforms comprising of the conventional short-read Illumina sequencing and third generation Oxford Nanopore Technology (ONT) long-read sequencing. Two samples were chosen (one from each Orang Asli group) labelled as JH16 (Jehai) and JA230 (Jakun) for sequencing on both platforms. By analysing sequencing depth against the number of assembled contigs, it is apparent that at 800 MB of Oxford Nanopore long-read or 20 GB of Illumina data per sample, the virome is not fully captured (see Supplementary Fig. S7 online). However, long-read sequencing readily assembles close to complete viral genomes as it was able to achieve greater N50 values and percentage of viral sequences in both samples compared to short-read sequencing (see Supplementary Fig. S8 and Table S5 online). This is likely due to short-read sequencing capturing only fragments of a genome in a single read, which often cannot be attributed to any specific viral genome. Long-read sequencing however, could theoretically cover, for many of the viruses, an entire genome in a single read. Hence, the low sequencing depth of long-read sequencing is overcome by the improved coverage of individual viral sequences and so it appears that breadth of coverage outperforms depth of coverage in this study.

Viral taxonomic annotations performed using vConTACT2 (see Supplementary Fig. S10 online), found that short-read sequencing revealed greater viral diversity compared to long-read sequencing. However, the low sequence coverage of viral sequences may also be involved in skewed misassignments of viral annotation in short-read sequencing. In summary, currently both sequencing technologies should be used in combination to provide a greater perspective of the virome, as short-read technologies provide greater sequencing depth while long-read provides closer to complete viral genomes. Recent improvements in long-read sequencing, such as High-Fidelity sequencing on PacBio Sequel II Systems, may provide alternate methods for investigating viromes in any environment. However, this may often not be possible due to resource constraints.

## Conclusion

This is the first human gut virome study from Malaysia, utilizing ONT long-read sequencing. The key finding and contribution of this work is the development of an effective protocol for the analysis of the human gut virome. Long-read sequencing improved the ability to characterize human gut virome by capturing long continuous sequences of viral genomes. This allowed us to observe the diversity, and uniqueness of the viromes of Orang Asli populations in this study. This preliminary study of the human gut virome in Malaysia has developed the appropriate methods and techniques that will be required for future virome studies, enabling the more extensive characterization of the virome from different ethnic groups in Malaysia. Clearly, most information can be obtained by combining long and short read sequencing. However, if resource constraints are an important factor, then much more information about viromes can be obtained from long read sequencing compared to short read sequencing.

## Methods

### Sample collection

Ethical approval was provided by the Monash University Human Research Ethics Committee (MUHREC Project ID 1516 and 17,859), which is per the Declaration of Helsinki, and complied with the international and institutional standards. Orang Asli faecal samples were collected from two locations in Malaysia; Jakun Orang Asli from Bekok, Segamat and Jehai Orang Asli, from Belum, Perak. Jakun faecal samples were collected in stool containers by The South East Asia Community Observatory (SEACO) data collectors between May and August 2018 and kept on ice packs and subsequently stored in − 80 °C freezers. Jehai faecal samples were collected in stool containers during the field visit and stored initially on dry ice and subsequently at − 80 °C. Three females from each group were randomly selected for this study. Short read and long read sequencing was done on two samples, JA230 and JH16. Based on the information obtained, only long read sequencing was performed on another four samples, JA223, JA237, JH18, and JH36 due to resource constraints. All participants provided written informed consent.

### Mock virome

Bacteriophages *Escherichia virus T4* (Order: *Caudovirales*, Family: *Myoviridae*, Genus: *Tequatrovirus*), *Escherichia virus Lambda* (Order: *Caudovirales*, Family: *Siphoviridae*, Genus: *Lambdavirus*), and *Escherichia virus T3* (Order: *Caudovirales*, Family: *Autographiviridae*, Genus: *Teetrevirus*) were obtained as aqueous extracts. These were subsequently cultured and used as controls to verify the efficiency of isolation and extraction of viral DNA. Two different strains of *E. coli* acted as hosts for these bacteriophages. *E. coli* B was the host for T4 and T3, whereas *E. coli* W3350 was the host for Lambda phage. Plaque assays were used to determine the titre of these bacteriophages. Bacteriophages were diluted and mixed at approximately 1186 ng, 209 ng, and 84 ng for T4, Lambda, and T3 respectively.

### Sample processing and viral matter isolation

Approximately 3 g of stool samples were placed inside a 50 mL screw cap centrifuge tube and kept on ice in triplicate (Total: 9 g of stool per individual). Ice-cold PBS was used as negative controls, which yielded no detectable DNA concentrations on Nanodrop and Qubit. Ice-cold PBS (30 mL) was added to each tube that was then vortexed at high speed for 5 min and placed back on ice. Tubes were then centrifuged at 5000 rpm for 10 min at 4 °C and supernatant transferred to a clean 50 mL centrifuge tube. This step was repeated once more to remove large debris particles. The supernatant was then filtered twice through 0.45 μm syringe filter. Saline (0.5 M NaCl) and 10% w/v PEG-8000 powder was added to the filtered solution and it was then incubated at 4 °C for 16 h.

### Purification of viral matter

Following PEG precipitation, samples were centrifuged at 5000 rpm for 20 min at 4 °C and the supernatant discarded. The tubes and pellet were left to dry on a paper towel for 10 min. Pellets were then resuspended in 200 μl of PBS and transferred to sterile 1.5 mL microcentrifuge tubes. An equal volume of chloroform was added and mixed gently before centrifugation at 2500 rpm for 5 min at room temperature. The aqueous phase was transferred into a new and sterile 1.5 mL microcentrifuge tube. Subsequently, 40 μl of a solution containing 10 mM CaCl_2_ and 50 mM MgCl_2_ was added to the tubes followed by 10 U of DNase and 10 U of RNase A. The tubes were incubated at 37 °C for 2 h. Nucleases were deactivated with heat inactivation at 70 °C for 10 min before proceeding with viral DNA extraction.

### Virome DNA extraction

Purified viral particles were first lysed with 40 μg of Proteinase K and 20 μl of SDS, and incubated at 56 °C for 2 h. Next, 100 μl of QIAGEN Buffer RLT was added and incubated at 65 °C for 10 min. Lysed viral particles were cleaned twice with equal volume of phenol:chloroform:isoamyl alcohol, pH 8.0 (25:24:1), centrifuged at 8000×*g* for five minutes at room temperature and the aqueous phase collected. Lastly, DNA was purified with QIAGEN DNeasy Blood and Tissue Kit with slight modifications to the manufacturer’s protocol. These included elution in 60 μl of nuclease-free water, incubation for 1 min at room temperature and centrifugation at 6000×*g* for 1 min at room temperature.

### Library preparation and sequencing

Short-read sequencing on the Illumina NovaSeq platform using 2 × 150-bp paired end sequencing was performed by Macrogen, Korea. Libraries were prepared using Nextera XT DNA library preparation kit. Short-read sequencing was used to acquire deep sequencing.

Long-read sequencing was performed on the Oxford Nanopore MinION platform. The library preparation used the PCR Sequencing Kit (SQK-PSK004) (Oxford Nanopore, UK) and sequenced according to manufacturer’s protocol.

### Processing of short- and long-read sequences

Short-read sequences were trimmed using Trimmomatic with a minimum quality of 20 and reads below 36 bp were removed, followed by examination on FastQC. Long-read sequences were assessed on NanoPlot. Assembly of sequences were split into three main types; short-read only, long-read only, and hybrid assembly. Short-read only were assembled using sequences obtained from short-read sequencing by metaSPAdes with flags -meta^44^. Long-read only were assembled using long-read sequences obtained from long-read sequencing by metaFlye with flags -meta and -genome^19^. Hybrid assembly was performed using short-read and long-read sequences by both Hybrid metaSPAdes with flags -meta and -nanopore as well as metaFlye with flags -meta and -genome followed by Pilon^45^ correction. Reads were tested for bacterial contamination by using ViromeQC^21^ and human host contamination by mapping reads with Minimap2^46^ against human reference genome (GRCh38). Reads and assemblies were mapped onto metaFlye sequences using Minimap2^46^ for long-read, Bowtie2^47^ for short-read, and aligned with Mummer^48^ for metaSPAdes and metaSPAdes-hybrid. Mapping and alignments were visualized with IGV^49^ and Genome Ribbon^50^ respectively.

### Viral identification and characterization

DNA viruses were then identified from assembled contigs using VirSorter^20^, using flags (-virome) to specify that the sequences are of viral origin, (-db 1) a built-in database and (-cp) an additional database, Human Gut Virome Database (HGVD)^5^. VirSorter predicts genes from input sequences and searches for these predicted genes against databases of known viral genes. Input sequences with genes that are similar to viral genes (E-value 10^–04^), are classified as a viral sequence^20^. Contigs identified as viral by VirSorter were subsequently translated to protein sequences. To better characterize these viral contigs, vConTACT2^23^ was used to assign taxonomy using HGVD. vConTACT2 utilizes clustering method by grouping input reads into separate protein clusters and viral clusters and compares between them to determine an accurate taxonomic assignment^23^. An in-house script was used to extract viral contigs with their respective assigned taxonomy from vConTACT2. Additionally, Genome Detective Virus Tool (GDVT) was used as an alternate approach for viral characterization and putative host prediction^24^. Auxiliary metabolic genes (AMG) were predicted using an existing workflow (https://dx.doi.org/10.17504/protocols.io.btv8nn9w) through a combination of tools including VirSorter2^51^, checkV^52^, and DRAM^33^.

### Statistical analysis

Data was analysed using Microsoft Excel or custom R scripts including functions from the following packages: Phyloseq^53^, Vegan^54^, ggplot2^55^. Viruses isolated were obtained from the NCBI Virus database, only viral order Caudovirales isolates were selected for subsequent analysis and split into continents of origin (last accessed on January 17, 2020). These were isolated from various sources including faeces and organs. The human gut virome database was analysed for viral abundance in different countries, including Cameroon (n = 29), China (n = 44), Finland (n = 56), Orang Asli (n = 6), Switzerland (n = 8), Uganda (n = 65), and USA (n = 279). Alpha and beta diversity was determined using R. Alpha diversity was examined using Vegan package in R by Chao1 and Simpson’s index and further analysed with pairwise Wilcoxon test (a non-parametric test to compare paired data). Beta diversity was examined using the Vegan package in R with Bray–Curtis dissimilarity matrix and principal coordinates analysis (PCoA) to estimate and visualize beta diversity in the samples. Beta diversity was further analysed using Vegan package; Adonis (permutational multivariate analyses of variance) and Pairwise Adonis (multi-level pairwise comparison). Dispersion was tested with the Vegan package Betadisper followed by ANOVA.

## Supplementary Information

Supplementary Information.

## Data Availability

The raw reads from this study are publicly available from National Center for Biotechnology Information (NCBI) sequence read archieve (SRA) database under Bioproject PRJNA747634.
