# Supplementary material for: The gut virome in two indigenous populations from Malaysia
Source: Sci Rep. 2022 Feb 3;12:1824. doi: 10.1038/s41598-022-05656-3 (PMC8813915; doi:10.1038/s41598-022-05656-3)
Supplement: Supplementary file 1 — Supplementary Information. [file 41598_2022_5656_MOESM1_ESM.docx]

**Supplementary Information for:**

The Gut Virome in Two Indigenous Populations from Malaysia

Chuen Zhang Lee^1^, Muhammad Zarul Hanifah bin Md Zoqratt^2^, Maude E. Phipps^3^, Jeremy J. Barr^4^, Sunil K. Lal^1^, Qasim Ayub^1,2,4^, Sadequr Rahman^1,5*^

^1^School of Science, Monash University Malaysia, 47500 Bandar Sunway, Selangor Darul Ehsan, Malaysia

^2^Monash University Malaysia Genomics Facility, 47500 Bandar Sunway, Selangor Darul Ehsan, Malaysia

^3^Jeffrey Cheah School of Medicine and Health Sciences, Monash University Malaysia, Subang Jaya, Malaysia

^4^School of Biological Sciences, Monash University, VIC, 3800, Australia

^5^Tropical Medicine & Biology Multidisciplinary Platform, Monash University Malaysia, Subang Jaya, Malaysia


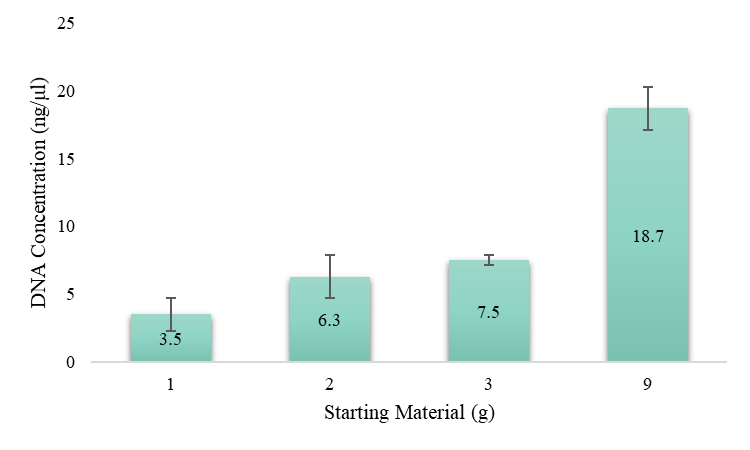


Supplementary Figure S1

DNA concentration (ng/μl) as determined by Qubit Fluorometer using different amounts of starting faecal material (1, 2, 3, and 9 grams) with Method 2.


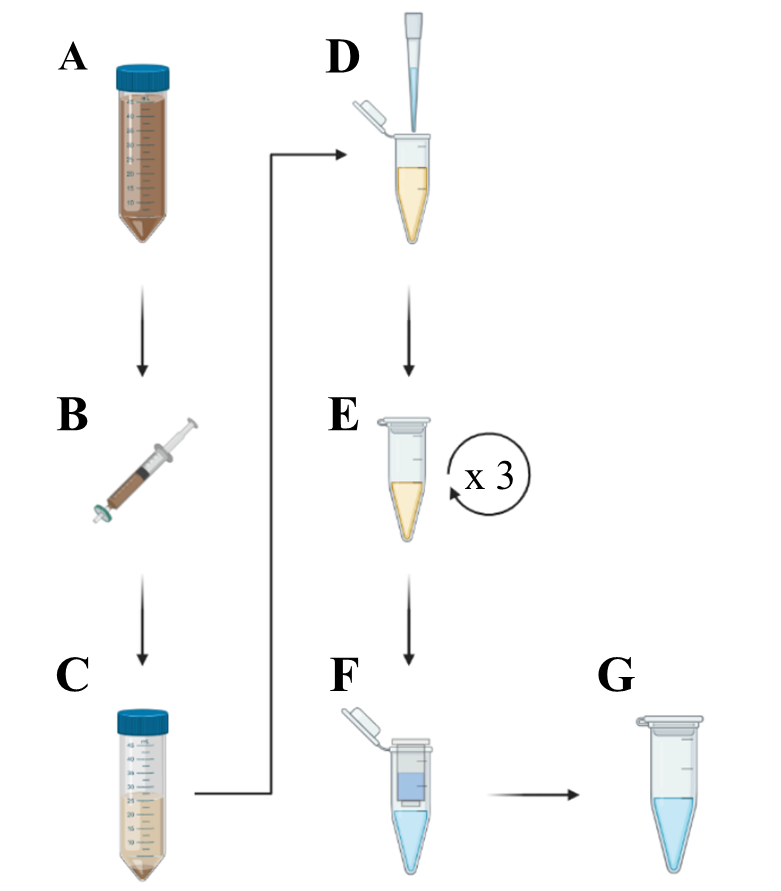


Supplementary Figure S2

Optimized Method 2 for virome DNA extraction. Designed on Biorender (https://biorender.com/). A: 3 g of faecal matter is mixed with 30 mL of PBS. B: Solution centrifuged and filtered to remove bacterial cells and debris. C: PEG added for precipitation of viruses. D: Pellet re-suspended and treated with DNase and RNase followed by twice of phenol:chloroform:isoamyl alcohol (25:24:1). E: Repeat steps A-D three times to create three tubes. F: Add three tubes of E one at a time through the DNeasy Blood and Tissue Kit column then followed by manufacturer’s protocol (Appendix B). G: Final elution of viral DNA.


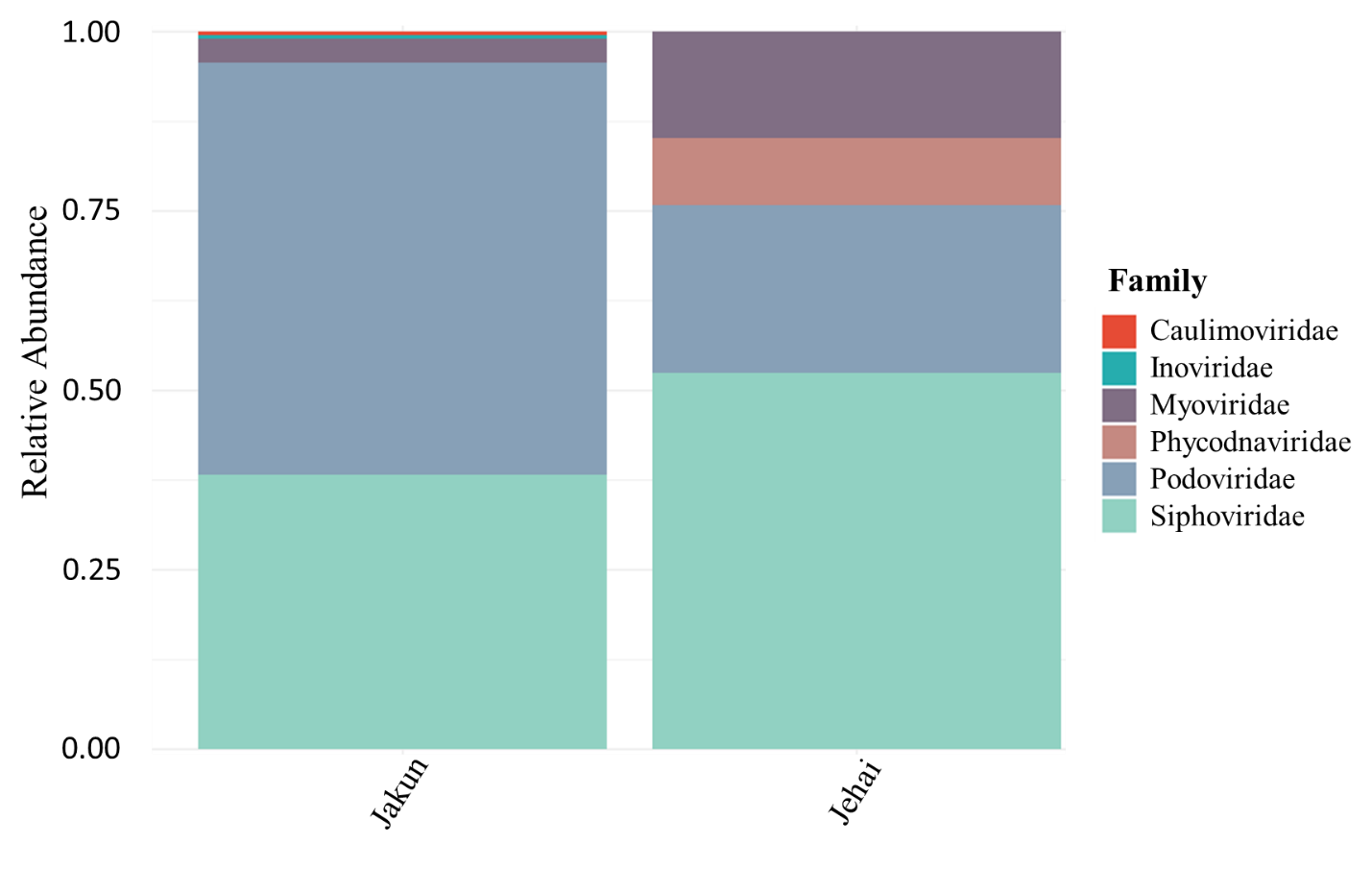


Supplementary Figure S3

Relative abundance of dominant viral families from the Jakun and Jehai Orang Asli. Taxonomy assigned from Genome Detective.

Supplementary Table S1 Pairwise Wilcoxon test of alpha diversity indexes between metagenomics viromes of Orang Asli, Malaysia and Other Countries.

|  | **Alpha Diversity Metrics** | |
| --- | --- | --- |
| **Pairs** | Chao1 | Inverse Simpson |
| Switzerland vs Malaysia | 0.66 | 0.89 |
| USA vs Malaysia | 0.92 | 0.13 |
| Finland vs Malaysia | 0.09 | 0.80 |
| Uganda vs Malaysia | 0.92 | 1.00 |
| Cameroon vs Malaysia | 1.00 | 1.00 |
| China vs Malaysia | 0.92 | 1.00 |

Significance code: < 0.001: ‘***’, < 0.01: ‘**’, < 0.05: ‘*’.

### Alpha-diversity Indexes

#### Chao1

The Chao1 index estimates the richness of species based on abundance. Richness is influenced by rare species with only a few representatives.

#### Simpson’s index

The Simpson’s index is a dominance index because it is weighted to dominant species. Therefore, diversity will not be affected by rare species with only a few representatives.

### Beta-diversity Ordination Method

#### Principal coordinate analysis (PCoA)

The PCoA begins with a dissimilarity matrix and assigns each species a location in a 2D space. PCoA finds the main solutions using eigenvalue decomposition.

#### Nonmetric multidimensional scaling (NMDS)

The NMDS is an iterative approximation algorithm and may find different solutions depending on the starting point. NMDS is a rank-based approach and can be used on any dissimilarity matrix.


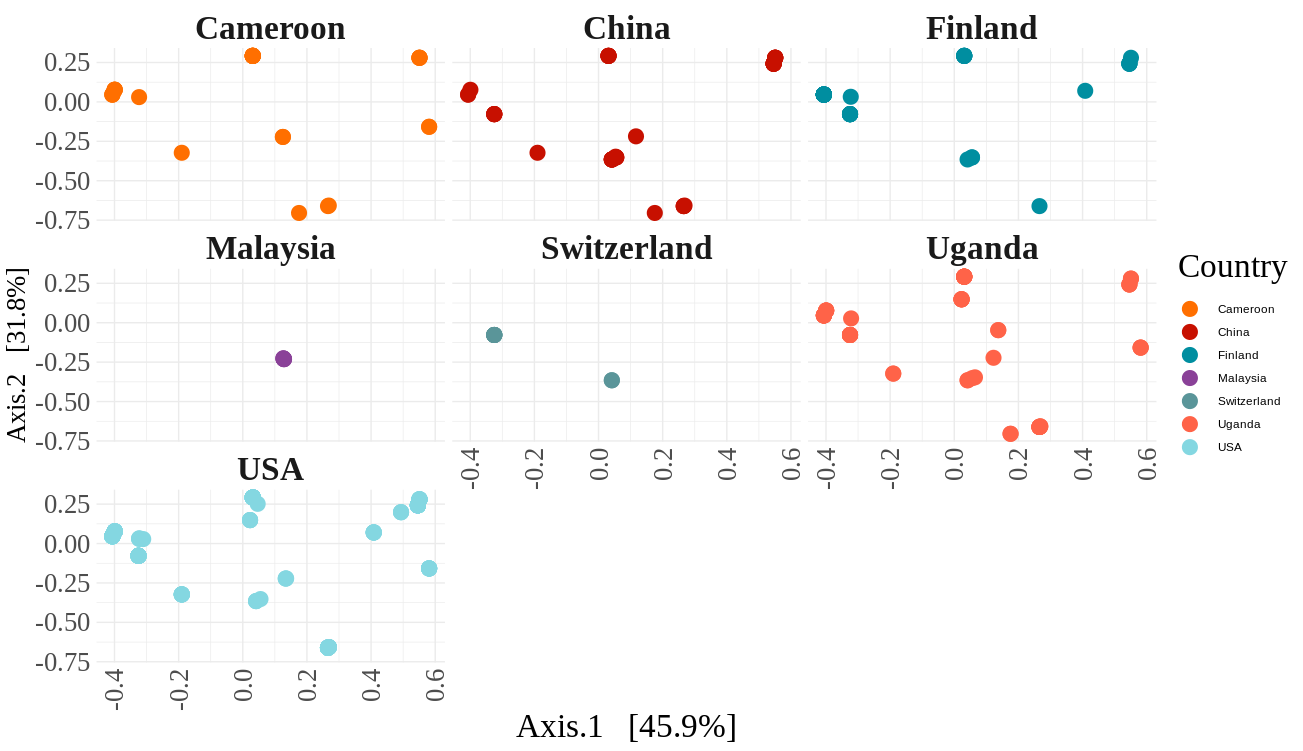


Supplementary Figure S4

PCoA plots using Bray-Curtis distance metrics split into countries


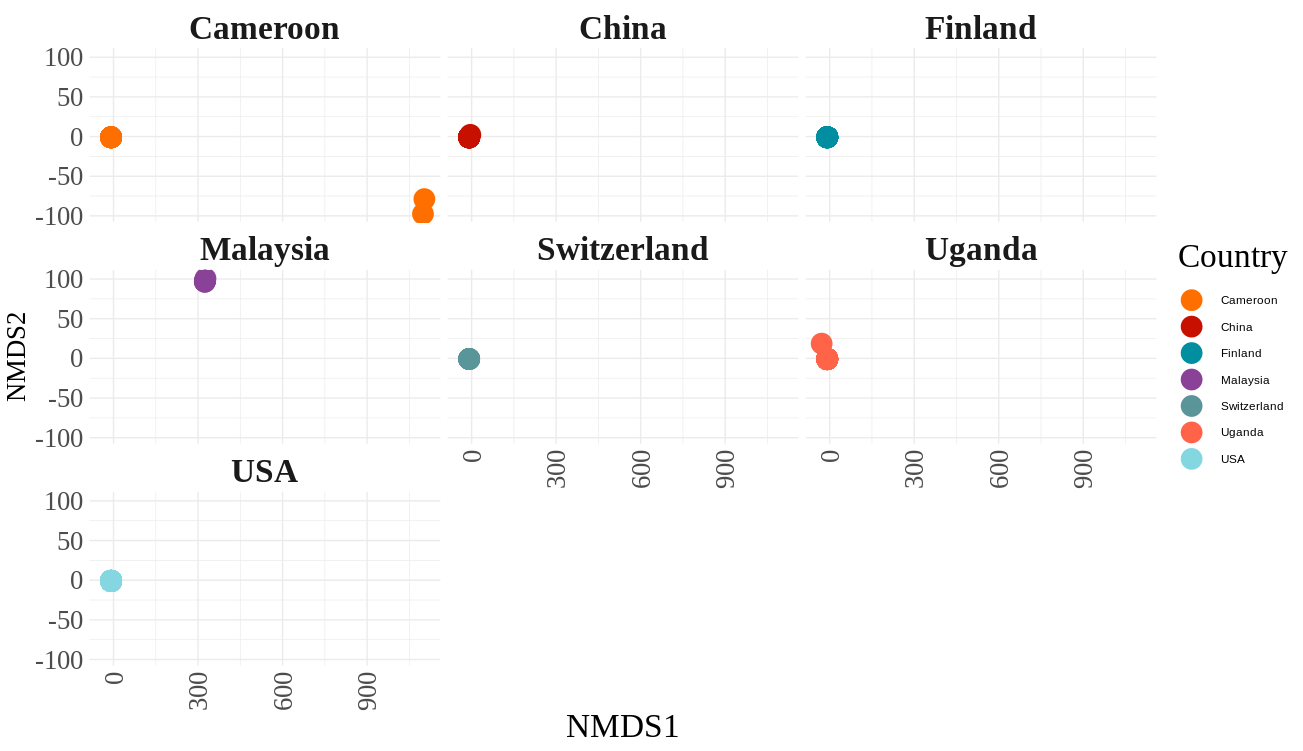


Supplementary Figure S5

**NMDS plots using Bray-Curtis distance metrics split into countries.**

Supplementary Table S2 Adonis (PERMANOVA) of all countries based on Bray-Curtis distance metrics.

| **Factor** | **Df** | **F-value** | **R^2^** | **p-value** |
| --- | --- | --- | --- | --- |
| Country | 6 | 8.14 | 0.09 | < 0.001 *** |

Significance code: < 0.001: ‘***’, < 0.01: ‘**’, < 0.05: ‘*’. R^2^ indicates effect size of factor.

Supplementary Table S3 Pairwise Adonis of country pairs.

| Pairs | Df | F-value | R^2^ | p-value | p-value adjusted |
| --- | --- | --- | --- | --- | --- |
| Switzerland vs Malaysia | 1 | 35.20 | 0.75 | < 0.001 *** | 0.02 * |
| USA vs Malaysia | 1 | 14.23 | 0.05 | < 0.001 *** | 0.02 * |
| Finland vs Malaysia | 1 | 20.05 | 0.25 | < 0.001 *** | 0.02 * |
| Uganda vs Malaysia | 1 | 12.87 | 0.16 | < 0.001 *** | 0.02 * |
| Cameroon vs Malaysia | 1 | 13.27 | 0.29 | < 0.001 *** | 0.02 * |
| China vs Malaysia | 1 | 9.23 | 0.16 | < 0.001 *** | 0.02 * |

Significance code: < 0.001: ‘***’, < 0.01: ‘**’, < 0.05: ‘*’. R^2^ indicates effect size of pairs. Malaysia represents the Orang Asli group from the current study. Pairs between other countries were not shown.

Supplementary Figure S6

Supplementary Table S4 AMG Module of Virome from Orang Asli Population.

|  | Samples | | | | | |
| --- | --- | --- | --- | --- | --- | --- |
| AMG Modules | JH16 | JH18 | JH36 | JA223 | JA230 | JA237 |
| Ribosome, bacteria | 34 | 0 | 0 | 0 | 0 | 0 |
| Pyrimidine deoxyribonuleotide biosynthesis | 25 | 6 | 1 | 0 | 0 | 3 |
| Ribosome, archaea | 17 | 0 | 0 | 0 | 0 | 0 |
| Glycosyl Transferases | 7 | 0 | 0 | 0 | 0 | 0 |
| Glycoside Hydrolases | 6 | 0 | 0 | 0 | 0 | 0 |
| Methionine degradation | 5 | 0 | 0 | 0 | 0 | 0 |
| Cysteine biosynthesis | 3 | 0 | 0 | 0 | 0 | 0 |
| Endopeptidases | 3 | 0 | 0 | 0 | 1 | 0 |
| Membrane-inserted endopeptidases | 3 | 0 | 0 | 0 | 0 | 0 |
| Gluconeogenesis | 2 | 0 | 0 | 0 | 0 | 0 |
| Glycolysis | 2 | 0 | 0 | 0 | 0 | 0 |
| Reductive citrate cycle | 2 | 0 | 0 | 0 | 0 | 0 |
| Dipeptidases | 2 | 0 | 0 | 0 | 0 | 0 |
| Exopeptidases that hydrolyse alpha-aspartyl bonds | 2 | 0 | 0 | 0 | 0 | 0 |
| Cell division transport system | 2 | 0 | 0 | 0 | 0 | 0 |
| NitT/TauT family transport system | 2 | 0 | 0 | 0 | 0 | 0 |
| Carbohydrate Esterases | 1 | 0 | 0 | 0 | 0 | 0 |
| Phosphate acetyltransferase-acetate kinase pathway | 1 | 0 | 0 | 0 | 0 | 0 |
| Galactose metabolism | 1 | 0 | 0 | 0 | 0 | 0 |
| Methanogenesis | 1 | 0 | 0 | 0 | 0 | 0 |
| Bacterial LD-carboxypeptidases | 1 | 0 | 0 | 0 | 0 | 0 |
| Cysteine endopeptidases | 1 | 0 | 0 | 0 | 0 | 0 |
| Exopeptidases | 1 | 0 | 0 | 0 | 0 | 0 |
| Isoleucine biosynthesis | 1 | 0 | 0 | 0 | 0 | 0 |
| alpha-2-macroglobulin | 1 | 0 | 0 | 0 | 0 | 0 |
| Metallo-endopeptidases | 1 | 0 | 0 | 0 | 0 | 0 |
| ABC-2 type transport system | 1 | 0 | 0 | 0 | 0 | 0 |
| Putative ABC transport system | 1 | 0 | 0 | 0 | 0 | 0 |

Percentage of each putative viral hosts among Jakun and Jehai.


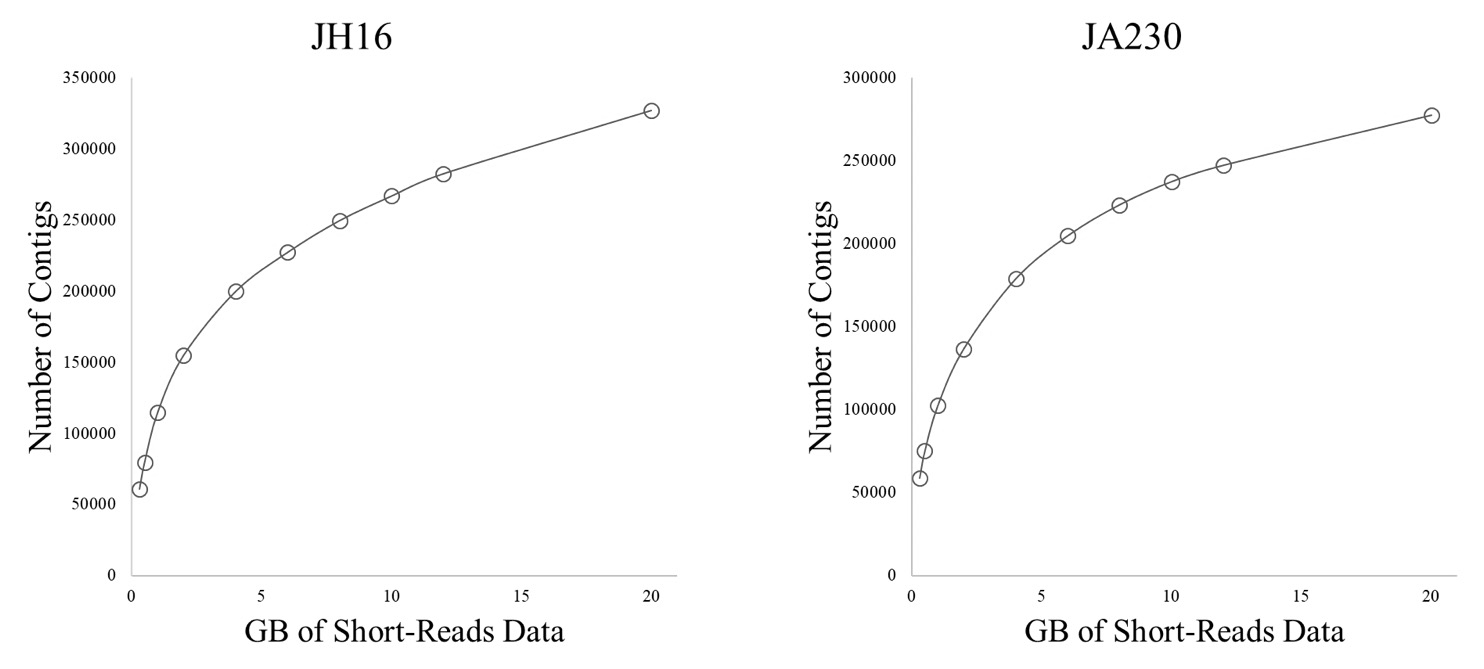


Supplementary Figure S7

Virome richness. Comparing number of contigs against depth of sequencing on samples JH16 and JA230.

To compare sequencing platforms of the conventional Next Generation Sequencing (NGS) and Oxford Nanopore Technology (ONT) long-read sequencing, two samples were chosen (one from each Orang Asli group) labelled as JH16 (Jehai) and JA230 (Jakun). By analysing sequencing depth against number of assembled contigs, it is apparent that at 800 Mbp of Oxford Nanopore long-read or 20 GB of NGS data, the virome is not fully captured (Figure S7). This may be attributed to the high variation in viral genomes due to high gene mutations in viruses. Therefore, we propose that future studies should increase the sequencing depth when analysing the gut virome.

Sequences obtained through assembly were compared between sequencing platforms NGS and long-read sequencing (Figure S8). The comparison of sequencing platform was divided into metaspades (NGS or short-read only), metaspades-hybrid (NGS polished by long-read sequences), metaflye (long-read only), and metaflye-corrected (long-read polished by short-read sequences). The total length of viral sequences and number of viral sequences were found to be larger through NGS platform compared to long-read sequencing, attributed to the large NGS sequencing depth. Contrastingly, the percentage of viral sequences were larger amongst long-read sequencing platform; JH16: 60% in metaflye and 37% in metaflye-corrected compared to 3% in metaspades 4% in metaspades-hybrid and JA230: 56% in metaflye and 28% in metaflye-corrected compared to 2% in both metaspades and metaspades-hybrid. The length of individual viral sequences was also compared, with results suggesting that long-read only and corrected-long-read had larger sequence lengths compared to short-read only and hybrid-short-read. Overall, this suggests the benefits of using the ONT sequencing platform in comparison to NGS. Despite improvements in metaspades-hybrid compared to metaspades, both assembly methods displayed low viral percentages. Surprisingly, metaflye-corrected had lower identified viruses than metaflye, potentially indicating a strength in using long-read individually to characterize viral metagenomes.

To better illustrate the coverage of genomes, short-read and long-read as well as sequences assembled by metaspades and metaspades-hybrid were compared with sequences assembled through metaflye (Figure S9). Three of the largest 20 sequences from each sample were randomly selected to be demonstrated in Figure S9, finding that long-read (nanopore) adequately covered the entire viral sequence as expected. Perhaps unsurprisingly, short-read (illumine) displayed uneven coverage of the viral sequences, which could also be observed in metaspades sequence alignments as there are many gaps within the viral sequence. Interestingly, viral sequence 230 from JH16 had good coverage in short-read, reflected in the perfect alignment of metaspades with the viral sequence. Additionally, the use of metaspades-hybrid improved the sequence coverage by generating larger sequences. Therefore, suggesting the strength of using long-read sequencing in viral metagenome assembly.

Supplementary Figure S8

Viral composition comparison between sequencing platforms. Jitter plot indicating sequences obtained through specified assembly program according to the following; short-read only – metaspades, short-read polished by long-read – metaspades_hybrid, long-read only – metaflye, long-read corrected by short-read – metaflye + corrected.

Supplementary Table S5 Metadata of viral sequences.

|  | JA230_metapades | JA230_metaspades_hybrid | JA230_metaflye | JA230_metaflye_corrected | JH16_metaspades | JH16_metaspades_hybrid | JH16_metaflye | JH16_metaflye_corrected |
| --- | --- | --- | --- | --- | --- | --- | --- | --- |
| No. of viral sequences | 363 | 374 | 104 | 52 | 940 | 945 | 461 | 564 |
| % of viral sequences | 2% | 3% | 56% | 28% | 3% | 4% | 60% | 37% |
| Median length of viral sequences (bp) | 6103 | 6325 | 21964 | 23716 | 5989 | 6976 | 25972 | 27677 |


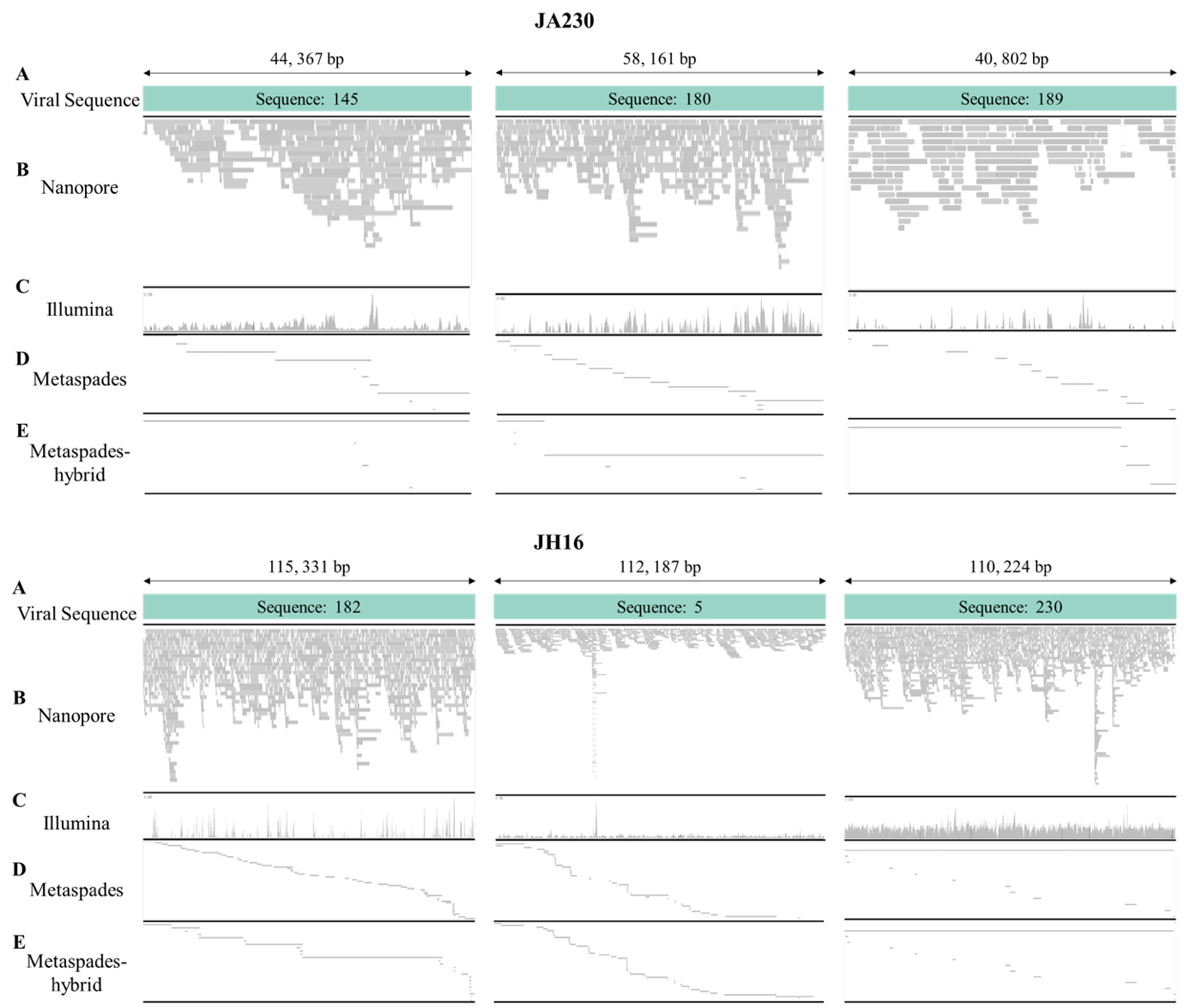


Supplementary Figure S9

Coverage of three out of the 20 largest viral sequences in each sample. A. Viral sequence assembled through metaflye using long-read only. B. Long-read sequencing read coverage on the viral sequence. C. Illumina read coverage on the viral sequence. D. Metaspades assembly sequence coverage on the viral sequence. E. Metaspades-hybrid assembly sequence coverage on the viral sequence.

Viral annotations were performed with vConTACT2 as the analysis workflow was mirrored across short-read and long-read sequencing. Both sequencing platforms were compared (metaspades for NGS and metaflye for long-read) (Figure S10), and it supports viral identification findings that sequencing with NGS captured a deeper virome compared to Oxford Nanopore, likely due to the larger sequencing depth on the NGS platform. In addition, hybrid assemblies in sample JH16, demonstrated a shift in abundance from metaflye to metaflye-corrected. A careful inspection of viral sequences that were taxonomically assigned in both metaflye and metaflye-corrected suggests that additional sequences were assigned after correction, thereby insinuating the importance of accurate assembly data in taxonomic assignments. Despite that, relative abundance of viral taxonomy did not change in sample JA230 between metaflye and metaflye-corrected, which could be attributed to the dominance of *Podoviridae* family in both short- and long-read. This demonstrates that importance of having an increased sequencing depth to fully capture the viral metagenome. On the other hand, long-read sequencing readily assembles close to complete viral genomes as it was able to achieve greater N50 values in both samples (JH16: 48517, JA230: 26686) compared to N50 values of NGS sequencing (JH16: 2893, JA230: 2080). In summary, ideally both sequencing technologies should be used when examining the virome, as NGS provides greater sequencing depth while long-read provides closer to complete viral genomes. Therefore, a combination of both factors would provide researchers a greater perspective on the virome. Nonetheless, relying on either sequencing platforms are beneficial in their own right, and should continue to be used in virome analysis.

Supplementary Figure S10

Comparing Sequencing Platforms and Assemblers on Samples JH16 and JA230 using metaspades, metaspades hybrid, metaflye, and metaflye corrected with pilon. Metaspades assembles using short-read only. Metaspades hybrid assembles using short-read and gap closure with long-read sequences. Metaflye assembles using long-read only. Metaflye corrected assembles using long-read then is error corrected with Pilon.

**Supplementary Figure S11**

Betadisper of countries. A. plot of betadisper. B. anova of betadisper. C. tukeyHSD of betadisper based on country pairs.
